# Predictive Value of Serum *HMGB1, NF-κB*, and *IL-17* Gene Expression in Acute Pancreatitis Outcomes

**DOI:** 10.3390/diagnostics15172160

**Published:** 2025-08-26

**Authors:** Milan Pantelić, Danijela Cvetković, Jovana Jovankić, Ivan Soldatović, Maša Pantelić, Miloš Dujović, Tamara Vučinić, Aleksandar Cvetković

**Affiliations:** 1 Department of Radiology, Zvezdara University Health Center, 11000 Belgrade, Serbia; 2 Department of Genetics, Faculty of Medical Sciences, University of Kragujevac, Svetozara Markovića 69, 34000 Kragujevac, Serbia; 3 Department of Biology and Ecology, Faculty of Science, University of Kragujevac, Radoja Domanovića 12, 34000 Kragujevac, Serbia; 4 Institute for Medical Statistics and Informatics, School of Medicine, University of Belgrade, 11000 Belgrade, Serbia; 5 Department of Gastroenterology and Hepatology, Zvezdara University Health Center, 11000 Belgrade, Serbia; 6 Department of General Surgery, University Clinical Center Kragujevac, 34000 Kragujevac, Serbia; 7 Department of Surgery, Faculty of Medical Sciences, University of Kragujevac, Svetozara Markovića 69, 34000 Kragujevac, Serbia

**Keywords:** acute pancreatitis, gene expression, biomarkers, *HMGB1*, *NF-κB*, *IL-17*

## Abstract

**Background/Objectives**: This study investigated the gene expression levels of *High Mobility Group Box 1 (HMGB1)*, *nuclear factor kappa B (NF-κB)* and *interleukin-17 (IL-17)* in the serum of patients with acute pancreatitis (AP) and analyzed the correlation of these three with the severity of AP, local and systemic complications, transfer to intensive care unit (ICU) and death. **Methods**: AP was diagnosed and stratified according to the revised Atlanta classification. The diagnosis of AP requires two of the following three features: abdominal pain (acute onset of persistent severe, epigastric pain often radiating to the back); serum lipase/or amylase activity at least three times higher than normal; characteristic findings of AP on computed tomography or abdominal ultrasonography. **Results**: This study confirmed that *NF-kB* is a significant marker of AP severity, as well as for ICU transfer, and correlates with acute respiratory distress syndrome (ARDS), while *IL-17* is shown as a significant marker of systemic complications (pleural effusions, ARDS, and renal failure). *HMGB1* correlates with pancreatic necrosis, systemic inflammatory response syndrome, and ICU transfer. **Conclusions**: Over the past years, the role of *HMGB1*, *NF-kB*, and *IL-17* in the pathogenesis of AP has been under intense scrutiny, and they have been proposed as prognostic biomarkers for AP severity, poor prognosis, and death outcome. The advantage of this research is that changes in gene expression can be detected before the increase in serum concentrations of these biomarkers, and it allows early prediction of a severe form of AP, as well as the development of complications.

## 1. Introduction

Acute pancreatitis (AP) is the most common gastrointestinal disease of highly variable severity, ranging from mild cases with low mortality to severe cases with high mortality. The revised Atlanta classification system has classified AP into mild, moderately severe, and severe. Mild AP was defined by the absence of local or systemic complications. Moderately severe AP was defined by the presence of transient organ failure, local complications, or exacerbation of co-morbid diseases. Severe AP was defined by persistent organ failure for more than 48 h [1,2,3]. Organ failure was defined as a score of 1 or more for one of the following features using the modified Marshall scoring system: (1) respiratory failure, defined as PaO_2_:FiO_2_ levels of 300 mmHg or less; (2) renal failure, defined as sCr level of at least 1.9 mg/dL and (3) shock, defined as systolic blood pressure of less than 90 mmHg and unresponsive to fluid therapy [1]. More than 80% of AP cases are mild and resolve without complications. In 20% of cases, it can be severe and complicated by major morbidity or mortality [1,2,3]. Early prediction of the severity and outcome of AP is important for the start of aggressive therapy, which is why the effectiveness of multifactorial scores and biomarkers of prognosis has been tested in recent years. Various multifactorial scoring systems (Ranson’s score, Bedside Index for Severity in AP-BISAP, The Acute Physiology and Chronic Health Evaluation-APACHE II score, and others), as well as numerous biomarkers (*C-reactive protein-CRP*, *procalcitonin-PCT*, various cytokines), are used to assess the severity of AP [4,5]. Limitations of these scoring systems include a delay in complete scoring, where it takes 48 h to complete the Ranson and Glasgow scoring systems, and the APACHE II score is very challenging to calculate.

Ranson’s score represented a major advance in evaluating the severity of AP but has the disadvantage of requiring a full 48 h to be completed (five parameters are measured upon admission to the hospital, and the other six in the next 48 h) [6]. APACHE-II can be administered on any day, and was initially designed to predict intensive care unit (ICU) survival.

BISAP can be calculated in the first 24 h after admission based on: urea nitrogen (BUN) (≥25 mg/dL), presence of impaired mental status (disorientation or other disturbance in mental status), presence of systemic inflammatory response, age ≥ 60 years, and presence of pleural effusion [1,7]. Systemic inflammatory response syndrome (SIRS) is defined by the presence of ≥2 of the following criteria: heart rate ˃ 90 beats/min, respiratory rate ˃ 20/min or pCO_2_ ˂ 32 mmHg, temperature ˃ 38 °C or ˂36 °C, and white blood cell count ˃ 12,000 or ˂4000 cells per mm^3^ or ˃10% immature neutrophils [8]. Baltazar et al. developed the Baltazar score for the estimation of the severity of AP based on computed tomography (CT) findings such as pancreatic size, the presence of pancreatic inflammation and peripancreatic fat, and the presence of peripancreatic fluid collections [4,9]. In 1990, they also developed the CT severity index (CTSI), combining the original Balthazar score with the presence of pancreatic necrosis [9]. In 2004, Mortele et al. formulated the modified CTSI (mCTSI), which is easier to calculate and correlates more closely with the outcome of AP, such as the occurrence of infections, organ failure, the need for surgical or percutaneous intervention, the length of hospital stay, and death [9,10]. The American Gastroenterological Association guidelines recommend that a CT scan be performed three days after the onset of illness, while the most recent studies recommend that it be done between 5–7 days [11].

The pathogenesis of AP has been investigated worldwide, and numerous studies have been published; however, the precise mechanism of pathogenesis remains unclear. The biggest barrier in the research of pathogenesis is the rapid and unpredictable course of the disease, as well as the relative inaccessibility of pancreatic tissue. Various studies have shown that multiple inflammatory mediators are involved in the pathogenesis of AP, among which are cytokines, adhesion molecules, chemokines, complement, neuropeptides, and others. Correlation between disease severity and level of the cytokines *IL-1*, *IL-6*, *IL-8*, *IL-10*, *IL-12*, *IL-17*, *TNF-α*, and *INF-γ* has been demonstrated [12,13,14,15,16,17,18,19]. Premature activation of trypsin in the pancreatic parenchyma as a central event in pancreatic tissue, autodigestion, and subsequent local and systemic inflammation is currently the most accepted theory. During the initial phase of AP, trypsinogen activation occurs, and *trypsinogen activation peptide (TAP)* is released, which initiates an enzymatic cascade leading to acinar cells’ autodigestion. Damaged cells release molecules known as *Damage-Associated Molecular Pattern (DAMP)*, which are recognized by cells of the immune system using *Pattern Recognition Receptors (PRR)*. *High Mobility Group Box 1 (HMGB1)*, *nucleosomes*, *DNA*, and *adenosine triphosphate (ATP)* have been identified as *DAMPs*. As a result of *DAMP* recognition, immune cells release inflammatory mediators such as cytokines (*IL-6*, *IL-10*, *IL-1β*, *TNF-α*) and chemokines (*IL-8*, *monocyte chemoattractant protein 1-MCP1*) that play a role in the mobilization of dendritic cells, monocytes, platelets, and neutrophils [20,21,22]. Also, studies have shown that high levels of *HMGB1*, *D-dimer*, *IL-1*, and *IL-17* are associated with a severe form of AP [4,13]. Previous experimental murine studies have shown that activation of the transcription *factor nuclear factor kappa B (NF-κB)* plays an important role as an early and central event in the pathogenesis of AP [7,23,24], which could represent the potential therapeutic target.

*HMGB1*, originally identified as a DNA-binding protein, is a protein that is expressed in various cell types, and its presence in the nucleus is necessary for transcriptional regulation and gene expression [20,25,26,27]. *TAP* has also been shown to favor the release of *HMGB1* from acinar cells. Investigations reported that serum HMGB1 levels are increased in patients with shock, hemorrhagic shock, acute lung injury, rheumatoid arthritis, and disseminated intravascular coagulation (DIC) [28,29]. Other studies have shown that plasmatic concentrations of *HMGB1* are significantly elevated in patients with severe AP compared to patients with a mild form of the illness [27,30]. *HMGB1* is secreted actively by living inflammatory cells such as macrophages/monocytes and is released passively from necrotic or injured cells [31]. In the extracellular space, *HMGB1* was also found to have the capacity to induce cytokines and activate inflammatory cells, and also binds to receptors such as *receptors for advanced glycation end products (RAGE)* and *to Toll-like receptor 4 (TLR4)*, linked to inflammatory processes. This implicates *HMGB1* as a proinflammatory mediator [20,32]. *HMGB1* serum level increases after 8–72 h, so it is classified as a late inflammatory mediator [26,27].

*NF-kB* belongs to a family of transcription factors known to regulate a broad range of processes such as immune cell function, proliferation and cancer, neuroprotection, and long-term memory. Activation of *NF-κB* during AP leads to the synthesis of numerous cytokines such as *TNF-α*, *IL-1β*, *IL-2*, *IL-6-*, and *IL-18*, various chemokines such as *IL-8*, *macrophage inflammatory protein (MIP-1)*, *MCP-1*, *platelet-activating factor (PAF)*, and adhesive molecules Activation of *NF-kB* is an early event in pancreatitis, paralleling trypsinogen activation in acinar cells. The relationship between key early events—trypsinogen activation and *NF-kB* activation—has been well-debated for over a decade, and various studies suggest that *NF-kB* activation is a key early event independent of trypsinogen activation and may be responsible for the progression of local and systemic inflammation [33,34,35,36]. Circulating cytokines (released from immune cells in a cascade reaction following activation of trypsinogen and the *NF-κB* signaling pathway) also act to further activate *NF-κB* within acinar cells as well as within other cells of the immune system, such as macrophages. Thus, *NF-kB* links the initial inflammation in the pancreas to systemic inflammation and plays a significant role in the further progression of inflammation. Therefore, unlike other inflammatory mediators that act only at a certain stage of AP development, *NF-κB* acts at different stages of AP progression from local inflammation to the development of SIRS [37].

*IL-17* is a cytokine that may also play an important role in the development of AP. *IL-17* is a characteristic cytokine of T helper 17 cells (Th17), but it can also be produced by other cells such as CD8+T cells, γδ T cells, and dendritic cells [38,39]. *IL-17* achieves its effect by binding to *IL-17 receptor*, which is expressed in various tissues and cells of the immune system. Numerous studies have shown that *IL-17* is present at sites of the inflammatory microenvironment and in synergistic interactions, enhancing inflammation induced by other cytokines, including *IL-1*, *IL-6*, *IL-8*, and *TNF-α* [40,41]. *IL-17* may amplify the inflammatory cascade during AP, contributing to the severity of AP because it enhances neutrophil recruitment to the site of inflammation by increasing the transcription of proinflammatory cytokines (such as *IL-1*, *IL-6*, *TNF-α*) and *neutrophil*-*attracting chemokine/cytokine* [38,42,43]. Also, some studies have confirmed the correlation between *IL-17* and AP, suggesting that *IL-17* may be a predictive marker for AP because it correlates with disease severity [14,15,44].

Based upon all these findings, this study investigated the gene expression levels of *HMGB1*, *NF-kB*, and *IL-17* in the serum of patients with AP and analyzed the correlation of these three with the severity of AP, local and systemic complications, transfer to ICU, and death. As far as we know, this is one of the few studies examining the correlation between serum *HMGB1*, *NF-kB*, and *IL-17* gene expression and the severity of AP in patients, given that most studies have been performed on animal models (murine model studies) and analyzed serum concentrations of these biomarkers.

To obtain an earlier prediction, we chose gene expression for this study, given that changes at the gene level can be detected before serum biomarker concentrations increase (which usually takes about 72 h).

## 2. Materials and Methods

### 2.1. Patient Selection

We conducted a prospective inception cohort observational study that consecutively enrolled adult patients diagnosed with AP and admitted to the Zvezdara University Clinical Center between March 2021 and April 2022. AP diagnosis and severity stratification were performed according to the revised Atlanta classification [2]. The diagnosis of AP required at least two of the following three criteria: (1) acute onset of persistent, severe epigastric pain often radiating to the back; (2) serum lipase and/or amylase activity at least three times the upper limit of normal; and (3) characteristic findings of AP on contrast-enhanced computed tomography or, less commonly, on transabdominal ultrasonography [2].

Patients were excluded if they had pancreatic carcinoma, incomplete medical documentation, or declined to provide informed consent.

The study protocol was approved by the Ethics Committee of the Zvezdara University Clinical Center, and written informed consent was obtained from all participants.

A total of 50 patients were finally included: 18 with mild AP, 24 with moderately severe AP, and 8 patients with severe AP classified according to the revised Atlanta classification. Demographic information of all enrolled patients was collected and recorded on admission. Clinical (blood pressure, respiratory rate, pulse rate, mental status) and laboratory parameters (white blood cell count, red blood cell count, neutrophil count, lymphocyte count, hemoglobin level, platelet level, hematocrit, renal function, hepatic function, electrolytes, *C-reactive protein*, *LDH*, glucose level, arterial blood gas) were assessed and recorded. The BISAP score was assessed on admission using the worst parameters available in the first 24 h. Also, the SIRS score was assessed on admission, while the Ranson score was calculated 48 h after admission. mCTSI was calculated in patients who underwent contrast-enhanced CT within 48 h of admission. All scores were calculated according to international criteria and analyzed.

**Figure 1:**
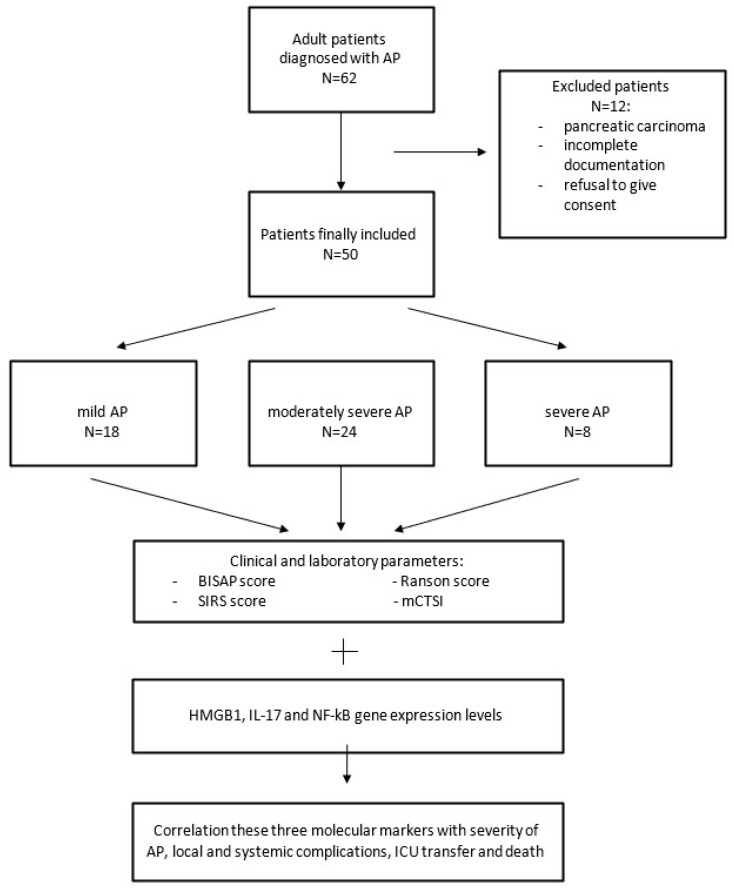
Flow chart of material and methods.

Specific disease outcomes whose association with molecular markers was investigated in this study were: the presence of local complications (ascites, pancreatic pseudocyst, pancreatic necrosis, and pancreatic abscess), systemic complications (pleural effusions, renal failure, and ARDS/need for mechanical ventilation-MV), transfer to ICU, and death (Figure 1). In addition to standard predictors of disease outcome, gene expression levels of molecular markers *HMGB1*, *IL-17*, and *NF-kB* from patient serum were also investigated using qRT-PCR technology.

### 2.2. Isolation of RNA from Patient Serum

Upon admission to the hospital, the patient’s blood was drawn and left undisturbed at room temperature for 15–30 min to clot. Afterwards, the coagulum was removed by centrifugation at 2000 rpm for 10 min in a refrigerated centrifuge. The samples obtained in this way were stored at −20 °C until the moment of use. Isolation of *RNA* from patient serum was performed with the Trizol Reagent. The 750 µL of Trizol was added to 250 µL of serum, the sample was homogenized with a pipette, inverted 5–8 times, and incubated at room temperature for 15 min. After the incubation time, 200 µL of chloroform was added to the samples, and the samples were inverted again and incubated for 5 min. This was followed by centrifugation at 12,000 rpm, 15 min, 4 °C. The upper aqueous phase was transferred to a new Eppendorf PCR tube, where 500 µL of isopropanol was previously added, and the sample was incubated for 10 min at room temperature, followed by centrifugation (12,000 rpm, 10 min, 4 °C). The isopropanol was poured off, and 1 mL of 75% ethanol was added to the *RNA* pellet, followed by another centrifugation (13,200 rpm, 7 min, 4 °C). Ethanol was added, and the *RNA* pellet was dried at room temperature in a laminar chamber for 10–15 min. Each sample of isolated *RNA* was resuspended in 15–20 µL of extremely pure water (PCR water). The purity of each sample was measured on a biophotometer. The absorbance ratio 260/280 nm was between 1.8 and 2.0, which is an indicator of purely isolated *RNA* without the presence of other components. The *RNA* samples thus obtained were aliquoted and stored at −80 °C until the experiments were performed.

### 2.3. Reverse Transcription

For the purposes of the reverse transcription reaction, it is necessary to translate the isolated single-stranded *RNA* into complementary *DNA*. In this experiment, the NG dART RT EURx kit is used, and the reaction mixture for each sample is prepared according to the manufacturer’s protocol. The individual reaction for each sample contained: 4 µL 5× NG cDNA Buffer, 1 µL random hexamer primer, 1 µL NG dART RT Mix, 2 µL RNA 500 ng, and 12 µL RNAse-free water. Samples were placed in an Eppendorf Mistercycler PCR machine and the machine was set according to the reverse transcription kit manufacturer’s protocol: the first cycle lasted 10 min at 25 °C, the second 50 min at 50 °C, and the third final cycle lasted 5 min at 85 °C. The obtained complementary *DNA* samples were stored at −80 °C until the experiments were performed.

### 2.4. Quantitative Polymerase Chain Reaction (qPCR)

Complementary *DNA* obtained by reverse transcription reaction was used for gene expression analysis. The SG/ROX qPCR Master Mix (2×) EURx kit was used for the purposes of this reaction. This kit contains all the necessary components for the reaction, except for a pair of primers and samples of complementary *DNA*. A reaction mixture contains: 10 µL SG/ROX qPCR Master MIX (2×), 0.5 µL Forward Primer, 0.5 µL Reverse Primer, 2 µL complementary *DNA*, and 7 µL RNAse-free water was made separately for each gene. The samples were placed in the Applied Biosystems^TM^ 7500 Real-Time PCR apparatus, and according to the manufacturer’s instructions: 1 cycle of initial denaturation for 20 min at 95 °C; 30 cycles of denaturation for 15 s at 94 °C; primer binding to *DNA* for 30 s at 60 °C, and polymerization for 30 s at 72 °C. The relative quantification of the expression of the investigated genes was obtained in relation to *β-actin* in the same sample. *β-actin* was used as a housekeeping gene in this experiment (primer sequences for β-actin, *IL-17*, *HMGB1*, and *NF-kB* genes are shown in Table 1). The following formula was used to calculate relative gene expression from serum samples of patients with AP:

ΔCt1 = Ct value of the tested gene in the sample;

ΔCt2 = Ct value of β-actin in the sample.

### 2.5. Statistical Analysis

Data distribution was assessed using the Shapiro–Wilk test and visual inspection of histograms and Q–Q plots to determine normality, which guided the selection of parametric or nonparametric statistical tests. Results are presented as count (%), mean ± standard deviation, or median (25th–75th percentile), depending on data type and distribution. Groups were compared using parametric (*t*-test, ANOVA) or nonparametric (Pearson chi-square, Fisher’s Exact test, Mann–Whitney U test, Kruskal–Wallis test) tests. Correlations between variables were assessed using Spearman’s rank correlation coefficient. Cut-off values were determined using ROC curve analysis. A *p*-value of <0.05 was considered statistically significant. All analyses were performed using SPSS version 29.0 (IBM Corp., Armonk, NY, USA) and R version 3.4.2 (R Foundation for Statistical Computing, Vienna, Austria).

## 3. Results

**Figure 2:**
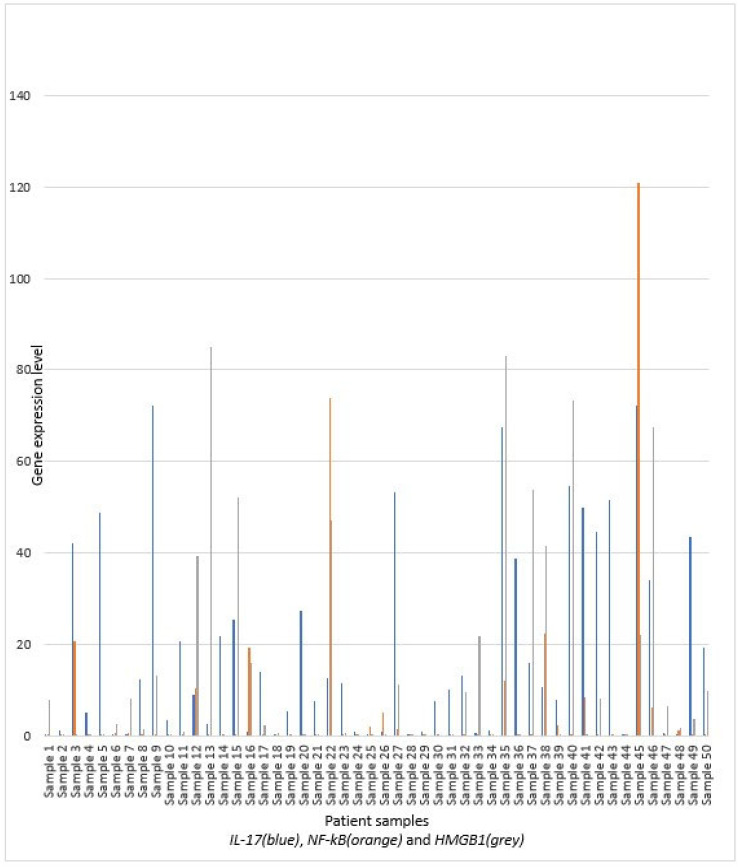
Gene expression levels of *IL-17*, *NF-kB* and *HMGB1* measured by qRT-PCR.

The study included 50 participants in total, the majority males (*n* = 32; 64%), with an average age of 62.6 ± 15.6 years. Duration of symptoms was 2 [1,2,3,4] days before hospitalization. The basic characteristics (gender, age, presence of comorbid conditions, smoking and cardiovascular disease, duration of symptoms before hospitalization) of patients are presented in Figure 2. Also, distribution of patients regarding the serum *IL-17*, *NF-kB*, and *HMGB1* gene expression levels is presented in Figure 2 and in Table 2. The median values of *IL-17* are higher in females and patients with significant comorbidities, especially with cardiovascular disease. Due to the high variability of *IL-17*, only the presence of comorbidities is near the conventional level of significance. Duration of symptoms before hospitalization has a positive correlation, but without significance. Younger participants have higher levels, but the *p*-value is near the conventional level of significance. The levels of *NF-kB* gene expression are similar across genders and age groups (with slightly higher levels in older individuals). No significant difference is observed in patients with comorbidities and smoking, although the levels are lower in smokers. *HMGB1* levels are higher in males and older patients, but without statistical significance. In patients with significant comorbidities, the *HMGB1* is lower, but also without significance.

Median values of *IL-17* are similar in patients with and without local complications—ascites, pancreatic pseudocyst, and necrosis. Patients with systemic complications and pleural effusion have significantly higher *IL-17* levels, compared to those without. Slightly higher levels are observed in patients with renal insufficiency, ARDS/MV (mechanical ventilation), and SIRS, but the sample size is insufficient for obtaining significance. No specific differences are observed in outcome variables (death and transfer to ICU) regarding *IL-17* gene expression levels.

Similar median values are observed in *NF-kB* regarding local complications—ascites, pancreatic pseudocyst and necrosis, and systemic complications. Gene expression in pleural effusion and renal insufficiency is higher but without significance (the variability is significantly higher). Patients with ARDS/MV showed a trend toward higher NF-κB gene expression compared to those without. Significantly higher levels are observed in patients transferred to the ICU, while the outcome of death is not significant.

*HMGB1* is higher in patients with all local and systemic conditions, but statistical significance is obtained only in pancreatic necrosis and SIRS. Although patients with a death outcome have higher *HMGB1* gene expression and transfer to the ICU, only the last is statistically significant (Table 3).

*IL-17* revealed no significant correlation with any of the presented scores, but an obvious trend was observed in the mCTSI score.

*NF-kB* revealed a significant correlation with Atlanta classification, while *HMGB1* correlates with pancreatic necrosis, mCTSI score, Ranson score, BISAP score, and Atlanta classification (not significant, but the trend is observed) (Table 4).

The ROC analysis of examined markers in correlation with significant conditions and outcomes is presented in Table 5. Similar to previous tables, IL-17 is shown as a significant marker of systemic complications. NF-κB is a significant marker of the severe form of the disease and ICU transfer, and shows a borderline association (trend) with ARDS. HMGB1 is a significant marker of pancreatic necrosis and SIRS, and probably ICU transfer.

## 4. Discussion

Pancreatitis is a clinically common serious disease of the digestive system and its incidence is increasing every year around the world, probably as a result of a combination of risk factors, such as obesity and gallstone disease. The overall mortality rate is 3% to 10%, but patients with the severe form are at an increased risk of death, with a mortality rate up to 50% [1,12]. Therefore, early differentiation between the mild and severe forms of AP is important.

Over the past years, various studies have also shown a positive correlation between serum *HMGB1* and the severity of the AP [13,27,45,46,47]. Scaffidi et al. demonstrated that apoptotic cells do not release *HMGB1* and do not induce an inflammatory reaction, as *HMGB1* is tightly bound to chromatin and will not be released into the extracellular matrix. By contrast, necrotic cells may passively release *HMGB1*. When cell necrosis occurs, cell membrane permeability increases and cell membrane integrity declines; therefore, *HMGB1* rapidly leaks from the cells and is easily detected [48]. Gao E et al. demonstrated in pancreatitis induced by cearulein that there was an association between *HMGB1* and necrotic cells of AP; *HMGB1* may be involved in inflammation and promote cell death by necrosis [49]. The results showed that with increased concentrations of caerulein, *HMGB1* protein expression gradually increased, which was consistent with an increase in necrotic cells [49]. In our study, we also showed that the serum *HMGB1* gene expression levels are higher in patients with necrotizing AP. Also, *HMGB1* gene expression is positively correlated with prognostic scores such as BISAP, Ranson, and mCTSI score, while the trend is observed in the Atlanta classification but without significance. Also, higher levels are revealed in patients with SIRS and ICU transfer. The findings in our study have indicated that *HMGB1* expression was increased in patients with necrotizing AP, patients with SIRS, and ICU transfer, suggesting that *HMGB1* can be regarded as an important cytokine mediator in the pathogenesis of severe AP, and it is proposed to eventually be an effective therapeutic target for these patients. Nevertheless, the exact mechanism by which the level of *HMGB1* is related to the development and progression of pancreatitis is not fully understood at present. Therefore, *HMGB1* inhibitors (neutralizing antibodies) may have therapeutic effects on AP. The inhibitors of *HMGB1*, such as ethylpyruvate and pyrrolidine dithiocarbamate, may inhibit *nuclear factor-κB* activation and reduce serum *HMGB1* levels; therefore, these inhibitors may protect against the dysfunction of a variety of organs [27,50,51].

This research showed that patients with systemic complications have significantly higher *IL-17* gene expression levels compared to those without, but without correlation with the severity of the AP (no statistically significant differences between mild, moderately severe, and severe AP). Also, *IL-17* revealed no significant correlation with any of the presented scores (Ranson, BISAP, mCTSI), although an obvious trend is observed in the mCTSI score. Therefore, *IL-17* gene expression may be used as a biomarker of systemic complications (pleural effusions, renal failure, and ARDS/need for MV) in patients with AP (moderately severe or severe form). *IL-17*, as a proinflammatory cytokine, has been found to be closely related to the pathophysiology of pancreatitis in recent years. To date, some researchers have confirmed the correlation between *IL-17* and AP and suggest *IL-17* as a predictive marker of the severity of the AP [13,14,15,39]. Vlachos et al. have shown that median values of *IL-17* were elevated during the first 24 h in patients with AP, whereas they were normal in the control group [52]. They also revealed that measurements of *IL-17* do not present statistically significant differences between mild and severe pancreatitis [52].

In this study, we identified that the serum *NF-kB* gene expression levels were higher in patients with severe AP; *NF-kB* revealed a positive correlation with the Atlanta classification/severity of the AP. *NF-kB* is increasingly gaining importance in recent years because its activation is an early and central event in the progression of inflammation in AP. Only a few studies have investigated *NF-kB* activation in human AP. Satoh et al. examined *NF-kB* activation in peripheral blood mononuclear cells (PBMC) of 45 patients with AP at admission and 14 days after the onset of the disease. They reported that the *PBMCs* from patients with AP showed higher levels of *NF-kB* activities than did those from control subjects [53]. Similar findings have shown O’Reilly et al. [54]. Also, these two studies [53,54] show no significant differences between patients with mild and severe AP. Alhan et al. showed in their animal model study that the measurement of p65 levels of *NF-kB* in *PBMCs* has no prognostic role during necrotizing AP in rats [51]. However, Huang et al. demonstrated in mouse models of the AP that the positive correlation between *NF-kB* activation and the severity of the AP [55]. In our study, patients with ARDS and need for MV have significantly higher *NF-kB* gene expression compared to those without, which can be explained by the activation of *NF-kB* in alveolar macrophages. Multiple cytokines are present in bronchoalveolar lavage fluid, so a common, proximal activation mechanism may operate in these settings. The proinflammatory cytokines (such as *TNF-α*, *IL-1*, *IL-6*, *IL-8*, *ICAM-1*) whose expression is increased in the lungs of patients with ARDS have binding sequences in their enhancer/promoter regions for *NF-kB* [33,56]. Moine et al. in their study confirmed that patients with ARDS had an increased activation of *NF-kB* in alveolar macrophages compared with the control group without acute lung injury [57]. *NF-kB* acts at different phases in the progression of local pancreatic inflammation to SIRS. Therefore, an inhibitor of *NF-kB* that acts at different levels in the inflammatory cascade could emerge as an ideal therapeutic agent. Specific *NF-kB* inhibition strategies (numerous substances derived from plants such as flavonoids, lignans, diterpenes, sesquiterpenes, polyphenols) have already been used in different cancers and other inflammatory conditions [58,59,60]. *Peroxisome proliferator activator receptor gamma (PPARγ) ligand*, *pyrrolidine dithiocarbamate (PDTC)*, *proteasome inhibitor*, and *calpain I inhibitor* have been shown to have direct inhibitory effects on *NF-kB* activation in AP [61,62,63]. Experimental/Preclinical studies that reported beneficial effects of different pharmacotherapeutic agents for AP were discussed by Jakampudi et al. [37].

Study limitations include a small number of patients enrolled, and because of that, the cut-off values for each discussed molecular marker cannot be established. Even though the number of patients in our study, especially those with severe AP, is small, and the results require further study and confirmation in a large prospective study, our data also indicate that the investigated biomarkers have the potential to be of clinical value in future AP stratification. Furthermore, due to the relatively small sample size, we were unable to perform a reliable multivariate analysis to adjust for potential confounding factors, which should be considered when interpreting the results.

## 5. Conclusions

In conclusion, in our study, we confirmed that *NF-kB* is a significant marker of AP severity, as well as for ICU transfer, and correlates with ARDS, while *IL-17* is shown as a significant marker of systemic complications (pleural effusions, ARDS, and renal failure). *HMGB1* correlates with pancreatic necrosis, SIRS, and ICU transfer.

Changes in gene expression can be detected before the increase in serum concentrations of these biomarkers, which is an advantage of this research because it allows early prediction of a severe form of AP, as well as the development of complications.

## Figures and Tables

**Table 1:** Primer sequences for *β-actin*, *IL-17*, *HMGB1*, and *NF-kB* genes.

Genes	Forward Primer	Reverse Primer
*β-actin*	5′-AAGCAGGAGTATGACGAGTCCG-3′	5′-GCCTTCATACATCTCAAGTTGG-3′
*HMGB1*	5′-GCTCAGAGAGGTGGAAGAC-3′	5′-CCAATGGATAAGCCAGGAT-3′
*NF-kapa B*	5′-ATGGCTTCTATGAGGCTGAG-3′	5′-GTTGTTGTTGGTCTGGATGC-3′
*IL-17*	5′-AGAGATATCCCTCTGTGATC-3′	5′-TACCCCAAAGTTATCTCAGG-3′

**Table 2:** Basic characteristics.

		N/Mean ± SD	*IL-17*	*p* Value	*NF-kB*	*p* Value	*HMGB1*	*p* Value
Gender	Male	32 (64%)	8.15 (16.04)	0.176 ^a^	0.223 (0.805)	0.686 ^a^	2.604 (11.543)	0.396 ^a^
	Female	18 (36%)	13.17 (47.32)		0.268 (3.007)		0.500 (8.277)	
Duration of symptoms before hospitalization								
(days)	Rho	2 (4)	0.190	0.196 ^b^	0.129	0.380	−0.135	0.362
Age	Rho	62.6 ± 15.6	−0.097	0.504 ^b^	0.051	0.723	−0.080	0.579
Age 65+	No	24 (48%)	14.18 (39.44)	0.077 ^a^	0.167 (1.248)	0.522 ^a^	2.623 (27.684)	0.426 ^a^
	Yes	26 (52%)	7.44 (14.3)		0.373 (1.230)		0.716 (8.26)	
Comorbid.	No	11 (22%)	0.8 (10.32)	0.081 ^a^	0.163 (11.341)	0.566 ^a^	4.215 (12.851)	0.266 ^a^
	Yes	39 (78%)	12.12 (35.58)		0.355 (1.224)		0.586 (10.247)	
Cardiovasc. Dis.	No	13 (26%)	3.41 (12.82)	0.196 ^a^	0.263 (0.343)	0.699 ^a^	3.752 (7.536)	0.420 ^a^
	Yes	37 (74%)	11.73 (35.58)		0.184 (1.224)		0.586 (10.247)	
Smoking	No	33 (66%)	11.73 (40.7)	0.112 ^a^	0.377 (4.345)	0.108 ^a^	2.861 (10.218)	0.532 ^a^
	Yes	17 (34%)	3.37 (14.23)		0.168 (0.316)		0.327 (4.115)	

Results are presented as median (IQR) or mean ± standard deviation; ^a^ Mann-Whitney U test; ^b^ Spearman correlation; Rho—Spearman correlation coefficient.

**Table 3:** Clinical findings.

		N	*IL-17*	*p* Value	*NF-kB*	*p* Value	*HMGB1*	*p* Value ^a^
Local complications	No	26 (52%)	11.7 (26.38)	0.969	0.359 (1.044)	0.846	0.416 (4.127)	0.162
	Yes	24 (48%)	6.57 (38.86)		0.170 (1.983)		4.581 (16.289)	
Ascites	No	27 (54%)	11.52 (14.88)	0.553	0.184 (1.044)	0.899	0.327 (5.096)	0.170
	Yes	23 (46%)	10.60 (41.34)		0.263 (2.742)		3.752 (16.243)	
Pancreatic pseudocyst	No	40 (80%)	11.59 (36.31)	0.528	0.360 (1.567)	0.115	0.675 (6.009)	0.127
	Yes	10 (20%)	3.99 (15.33)		0.113 (0.248)		9.005 (36.276)	
Pancreatic necrosis	No	40 (80%)	11.06 (35.54)	0.771	0.27 (0.852)	0.961	0.416 (4.611)	0.007
	Yes	10 (20%)	7.73 (17.40)		0.193 (3.027)		11.662 (58.823)	
Abscess †	No	49 (98%)	11.52 (35.19)		0.263 (1.224)		2.437 (10.218)	
	Yes	1 (2%)	4.58		0.015		0.049	
Systemic complications	No	29 (58%)	3.37 (17.4)	0.030	0.184 (0.393)	0.461	0.847 (8.249)	0.930
	Yes	21 (42%)	12.12 (34.12)		0.263 (3.015)		2.437 (10.191)	
Pleural effusion	No	32 (64%)	3.39 (22.22)	0.048	0.173 (0.41)	0.130	0.587 (7.926)	0.455
	Yes	18 (36%)	11.93 (34.12)		0.552 (11.309)		2.649 (16.278)	
Renal insuff.	No	37 (74%)	7.71 (35.3)	0.304	0.178 (0.557)	0.325	0.586 (7.604)	0.283
	Yes	13 (26%)	12.12 (8.26)		0.492 (3.015)		2.861 (15.93)	
ARDS/MV ^+^	No	45 (90%)	7.75 (26.87)	0.140	0.168 (0.669)	0.059	0.764 (8.26)	0.253
	Yes	5 (10%)	12.12 (38.75)		2.776 (2.57)		2.861 (7.881)	
Transfer to ICU	No	33 (66%)	10.60 (35.19)	0.767	0.167 (0.331)	0.015	0.143 (4.153)	0.004
	Yes	17 (34%)	11.66 (15.20)		1.279 (16.836)		7.692 (20.623)	
Death outcome ^+^	No	46 (92%)	9.17 (35.21)	0.436	0.173 (1.047)	0.132	0.806 (8.26)	0.256
	Yes	4 (8%)	11.89 (21.15)		1.634 (6.689)		6.378 (52.637)	
	Yes	4 (8%)	11.89 (21.15)		1.634 (6.689)		6.378 (52.637)	
SIRS ^+^	No	42 (84%)	8.15 (35.30)	0.204	0.181 (1.044)	0.594	0.675 (7.621)	0.069
	Yes	8 (16%)	13.85 (25.67)		0.378 (2.848)		7.864 (31.43)	

Results are presented as median (IQR); ^a^ Mann-Whitney U test was used in all analyses, ^+^ Exact test used; † Insufficient number for statistical analysis.

**Table 4:** Correlation with scores in use.

		N	*IL-17*	*p* Value	NF-kB	*p* Value	*HMGB1*	*p* Value
mCTSI								
pancreatic inflammation	Normal	22	12.35 (36.26)	0.404 ^b^	0.173 (1.047)	0.741 ^b^	0.138 (5.096)	0.186 ^b^
	Changed	6	13.28 (25.4)		0.488 (0.532)		0.675 (0.343)	
	Liquid collections	22	3.99 (17.31)		0.309 (2.685)		4.581 (16.278)	
pancreatic necrosis	No	40	11.06 (35.54)	0.223	0.27 (0.852)	0.200	0.416 (4.611)	0.018
	≤30%	7	0.8 (17.48)		0.091 (3.045)		7.692 (61.371)	
	>30%	3	16 (38.29)		2.776 (61.532)		16.434 (87.58)	
pancreatic necrosis ^+^	No	40	11.06 (35.54)	0.771 ^a^	0.27 (0.852)	0.961 ^a^	0.416 (4.611)	0.007 ^a^
	Yes	10	7.73 (17.4)		0.193 (3.027)		11.662 (58.823)	
extrapanc. comp.	No	24	12.35 (31.09)	0.698 ^a^	0.27 (0.386)	0.472 ^a^	0.24 (4.611)	0.200 ^a^
	Yes	26	9.58 (34.1)		0.22 (3.013)		3.307 (16.243)	
Score	Mild	23	13.17 (36.26)	0.828 ^b^	0.178 (1.047)	0.866 ^b^	0.143 (5.096)	0.065 ^b^
	Moderate	18	9.58 (26.87)		0.357 (0.613)		0.675 (16.277)	
	Severe	9	3.34 (15.65)		0.263 (3.027)		10.318 (11.025)	
Ranson score								
Heavy form ^+^	≤3	41	11.66 (36.26)	0.272 ^a^	0.263 (0.667)	0.455 ^a^	0.847 (7.592)	0.804 ^a^
	4+	9	3.37 (11.44)		0.178 (2.637)		2.903 (22.933)	
Mortality	2%	21	13.17 (32.54)	Rho −0.105	0.168 (0.296)	Rho 0.175	0.764 (4.072)	Rho 0.101
	15%	16	3.39 (39.25)		0.271 (2.118)		0.122 (6.048)	
	40%	11	7.75 (15.33)		0.167 (16.91)		16.377 (29.643)	
	100%	2	10.11 (3.1)		5.925 (10.865)		1.471 (1.933)	
Bedside index (mortality)	Low	40	10.60 (36.47)	0.550 ^a^	0.168 (0.566)	0.104 ^a^	0.586 (7.604)	0.131 ^a^
	High	10	11.66 (13.05)		1.279 (11.309)		5.409 (29.266)	
Atlanta classification	Mild	18	9.61 (35.66)	0.592 ^b^	0.373 (0.926)	0.036 ^b^	0.24 (5.079)	0.467 ^b^
	Moderate	24	9.17 (30.24)		0.097 (0.229)		1.768 (17.953)	
	Severe	8	11.89 (28.53)		2.028 (6.832)		4.135 (11.905)	
Atlanta Moderate/Severe	No	18	9.61 (35.66)	0.372 ^a^	0.373 (0.926)	0.394 ^a^	0.24 (5.079)	0.934 ^a^
	Yes	32	11.13 (30.24)		0.151 (1.985)		2.79 (14.607)	
Atlanta Severe ^+^	No	42	9.17 (35.21)	0.474 ^a^	0.167 (0.566)	0.044 ^a^	0.675 (8.26)	0.278 ^a^
	Yes	8	11.89 (28.53)		2.028 (6.832)		4.135 (11.905)	

Results are presented as median (IQR); ^a^ Mann-Whitney U test; ^b^ Kruskall Wallis test; ^+^ Exact test used.

**Table 5:** ROC analysis of *IL-17*, *NF-kB*, and *HMGB1*.

	Area	95% CI	*p* Value	Cut Off	Sn	Sp
IL-17						
Systemic compl.	0.681	0.535–0.828	0.030	3.99	0.810	0.552
Pleural effusion	0.670	0.521–0.820	0.048	3.99	0.833	0.531
NF-kB						
ARDS	0.760	0.613–0.907	0.059	0.488	0.800	0.711
ICU	0.711	0.540–0.883	0.015	0.488	0.647	0.788
Atlanta severe form	0.726	0.534–0.919	0.044	0.488	0.750	0.738
HMGB1						
Panc. Necrosis	0.777	0.594–0.961	0.007	2.835	0.900	0.675
ICU	0.754	0.617–0.891	0.004	0.154	0.941	0.545
SIRS	0.705	0.530–0.881	0.068	2.835	0.750	0.619

## Data Availability

The data that support the findings of this study are available from the corresponding author (M.P.) upon reasonable request.
